# Supplementary material for: Investigation of the Entry Pathway and Molecular Nature of σ1 Receptor Ligands
Source: Int J Mol Sci. 2023 Mar 28;24(7):6367. doi: 10.3390/ijms24076367 (PMC10094450; doi:10.3390/ijms24076367)

Legends to Supplementary Tables S1–S4 and S6–S8

Supplementary Table S1. Results of VS experiments against the 3D structure of *Hs*σ1-R in coordinate file 5HK1_A. Only hits whose calculated interaction energy with the target structure is ≤ 3.0 kcal/mol higher than that of the best hit are reported. Database ID: Database identifier. Compound name: Common name of the compound. Category: Category to which each compound is assigned. Ago-Ant: known σ1-R and/or σ2-R binder. Metab, FDA and World: molecules tagged as “metabolites+for sale”, “FDA approved+for sale” and “World-not FDA+ for sale” in the ZINC15 database (43). Ste_Lip, Sterols, Steroids, Androg, Estrog, Chol_P and Ergo_P: molecules belonging to the sterol_lipids, sterols, steroids, androgens, estrogens, cholesterol or ergosterol biosynthetic pathway and available for sale in the LIPID MAPS database (44). E_{calc} (kcal/mol): calculated interaction energy between each compound and the target protein. HB, CL, Contacts: Hydrogen bonds, unfavorable van der Waals contacts and overall contacts between each compound and the target protein, respectively.

Supplementary Table S2. Results of VS experiments against the 3D structure of *Hs*σ1-R in coordinate file 6DK1_A. Only hits whose calculated interaction energy with the target structure is ≤ 3.0 kcal/mol higher than that of the best hit are reported. All abbreviations are as in Supplementary Table 4->1.

Supplementary Table S3. Comparison between the results of VS experiments against the 3D structures of *Hs*σ1-R in coordinate files 5HK1 and 6DK1. Only hits whose calculated interaction energy with each target structure is ≤ 3.0 kcal/mol higher than that of the best hit are reported. All abbreviations are as in Supplementary Table 4->1.

Supplementary Table S4. Results of VS experiments against the yeast ERG2 molecular model. Only hits whose calculated interaction energy with the target structure is ≤ 3.0 kcal/mol higher than that of the best hit are reported. All abbreviations are as in Supplementary Table 4->1.

Supplementary Table S6. Results of VS experiments against the *Xl*σ1-R structure in coordinate file 7W2B. Only hits whose calculated interaction energy with the target structure is ≤ 3.0 kcal/mol higher than that of the best hit are reported. All abbreviations are as in Supplementary Table 4->1.

Supplementary Table S7. Results of VS experiments against the *Xl*σ1-R structure in coordinate file 7W2E. Only hits whose calculated interaction energy with the target structure is ≤ 3.0 kcal/mol higher than that of the best hit are reported. All abbreviations are as in Supplementary Table 4->1.

Supplementary Table S8. Comparison between the results of VS experiments against the 3D structures of *Xl*σ1-R in coordinate files 7W2B and 7W2E. Only hits whose calculated interaction energy with each target structure is ≤ 3.0 kcal/mol higher than that of the best hit are reported. All abbreviations are as in Supplementary Table 4->1.

Supplementary Table S5. Results of VS experiments against the 3D structure of *Hs*σ1-R in coordinate files 5HK1_A and 6DK1_A, for low affinity *Hs*σ1-R binders. Compound_name: Common name of compounds that are reported to bind human σ1R with the lowest affinity (i.e., Ki > 10,000 nM) in the PDSP Ki database. ZINC_ID: ZINC database identifier. Rank: Position in the E_{calc}-based ranking. E_{calc} (kcal/mol): calculated interaction energy between each compound and the target protein. Hb, Cl, Con: Hydrogen bonds, unfavorable van der Waals contacts and overall contacts between each compound and the target protein, respectively.

Compound_name	ZINC_ID	5HK1					6DK1				
		Rank	Ecalc	Hb	Cl	Con	Rank	Ecalc	Hb	Cl	Con
THC	ZINC000001530625	1992	-9.9	0	1	57	6378	-9.0	0	1	49
Naringenin	ZINC000000156701	2721	-9.6	0	0	47	5457	-9.3	0	0	39
Epigallocatechin	ZINC000003870336	4217	-9.1	0	0	48	7745	-8.6	0	0	45
Catechin	ZINC000000119978	4793	-8.9	0	0	42	5150	-9.4	0	0	38
Tasimelteon	ZINC000004392649	7334	-8.2	0	0	38	5216	-9.4	0	0	57
Tramadol(+-)	ZINC000000000853	7960	-8.0	0	0	51	9819	-8.0	0	0	33
Isoginkgetin	ZINC000003197535	10549	-7.4	0	1	41	11698	-7.5	0	3	126
Amentoflavone	ZINC000003984030	10565	-7.4	0	0	39	4641	-9.6	0	3	115
Morphine	ZINC000003812983	12409	-7.0	0	1	62	5813	-9.2	0	0	48
Oxymorphone	ZINC000003875483	14612	-6.6	0	4	73	8075	-8.5	0	0	42
Hydromorphone	ZINC000000402954	14991	-6.5	0	3	67	5154	-9.4	0	0	48
Oxycodone	ZINC000000403533	16601	-6.2	0	4	69	8689	-8.3	0	0	41

Supplementary Table S9. RMSD values calculated after optimal all-against-all structure superposition of *Xl*σ1R monomers superposition of all monomers with chain ID "A" in each PDB file. Residues used for the superpositions are the same used for intra-structure comparisons in the case of Cα atoms. Since residues at positions 179 and 203 are mutated in coordinate file with PDB ID 72WH with respect to all other coordinate files, residues included in all atoms superpositions were 31-178, 180-202 and 204-216, for a total of 2914 atoms. Med, Ave, St Dev, Min, Max: Median, average, standard deviation, minimum and maximum value. RMSD Cα and RMSD all: RMSD values calculated after optimal structure superposition of Cα and all atoms, respectively.

Inter-structure comparison, RMSD Cα							
	**7W2B**	**7W2C**	**7W2D**	**7W2E**	**7W2F**	**7W2G**	**7W2H**
**7W2B**	-						
**7W2C**	0.18	-					
**7W2D**	0.22	0.25	-				
**7W2E**	0.60	0.57	0.58	-			
**7W2F**	0.59	0.56	0.57	0.14	-		
**7W2G**	0.60	0.57	0.58	0.14	0.12	-	
**7W2H**	0.43	0.4	0.41	0.35	0.35	0.36	-

	**Med**	**Ave**	**St Dev**	**Min**	**Max**		
	0.41	0.41	0.18	0.12	0.60		

Inter-structure comparison, RMSD All atoms							
	**7W2B**	**7W2C**	**7W2D**	**7W2E**	**7W2F**	**7W2G**	**7W2H**
**7W2B**	-						
**7W2C**	0.37	-					
**7W2D**	0.42	0.33	-				
**7W2E**	1.05	1.04	1.03	-			
**7W2F**	1.05	1.04	1.04	0.26	-		
**7W2G**	1.05	1.04	1.04	0.28	0.23	-	
**7W2H**	0.85	0.78	0.79	0.72	0.74	0.74	-

	**Med**	**Ave**	**St Dev**	**Min**	**Max**		
	0.79	0.76	0.31	0.23	1.05		

Supplementary Table S10. 3D structures of *Xl*σ1-R available from the PDB. PDB ID: PDB identifier. Method: Experimental methods of 3D structure determination. X-ray: X-ray crystallography. Res. (Å): Resolution (angstrom). Chains: Chain identifier in the PDB file. A.a.: Amino acid residues in the protein. SOI: Ligand that is subject of investigation. E84: 2-morpholin-4-ylethyl 1-phenylcyclohexane-1-carboxylate; 88E: 4-[2-(5-methyl-1-naphthalen-2-yl-pyrazol-3-yl)oxyethyl]morpholine. Ligand: Ligand that is not a subject of investigation. BOG: octyl beta-D-glucopyranoside; Au: gold ion. Form: protein conformation. Mutations: Residues mutated with respect to the WT protein.

PDB ID	Method	Res. (Å)	Chains	a.a.	SOI	Ligand	Form	Mutations
7W2B	X-ray	3.20	A-L	1-221	-	BOG, Au	closed	-
7W2C	X-ray	3.33	A-L	1-221	E84	BOG, Au	closed	-
7W2D	X-ray	3.47	A-L	1-221	88E	Au	closed	-
7W2E	X-ray	3.56	A-L	1-221	-	-	open-like	-
7W2F	X-ray	3.10	A-L	1-221	E84	BOG	open-like	-
7W2G	X-ray	2.85	A-L	1-221	E84	BOG	open-like	-
7W2H	X-ray	3.80	A-X	1-221	88E	-	open-like	Y179C, Y203C

Supplementary Table S11. RMSD values calculated after optimal all-against-all structure superposition of *Xl*σ1R monomers within the same coordinate file. Residues 31-216 are used for all superpositions since they comprise the whole ligand binding domain and are present in all monomers. These comprise 186 Cα atoms, and 2938 and 2954 total atoms for PDB ID 7W2H and for all other PDB IDs, respectively. Med, Ave, St Dev, Min, Max: Median, average, standard deviation, minimum and maximum value. RMSD Cα and RMSD all: RMSD values calculated after optimal structure superposition of Cα and all atoms, respectively.

Intra-structure comparison, RMSD Cα						
**PDB ID**	**Chains**	**Med**	**Ave**	**St Dev**	**Min**	**Max**
**7W2B**	A-L	0.25	0.29	0.12	0.10	0.47
**7W2C**	A-L	0.24	0.31	0.13	0.12	0.49
**7W2D**	A-L	0.21	0.29	0.13	0.11	0.45
**7W2E**	A-L	0.16	0.15	0.04	0.07	0.22
**7W2F**	A-L	0.18	0.18	0.04	0.07	0.25
**7W2G**	A-L	0.15	0.15	0.03	0.06	0.22
**7W2H**	A-X	0.15	0.15	0.03	0.06	0.21

Intra-structure comparison, RMSD all atoms						
**PDB ID**	**Chains**	**Med**	**Ave**	**St Dev**	**Min**	**Max**
**7W2B**	A-L	0.64	0.63	0.14	0.29	0.87
**7W2C**	A-L	0.54	0.59	0.23	0.16	0.86
**7W2D**	A-L	0.52	0.57	0.26	0.16	0.84
**7W2E**	A-L	0.49	0.43	0.13	0.15	0.56
**7W2F**	A-L	0.52	0.52	0.07	0.22	0.61
**7W2G**	A-L	0.43	0.42	0.06	0.23	0.54
**7W2H**	A-X	0.21	0.21	0.04	0.08	0.33

Supplementary Figure S1. Root-mean-square deviation, calculated on the protein backbone of the whole trimer and of each monomer (MA, MB, and MC) with respect to the starting structure, as function of simulation time.

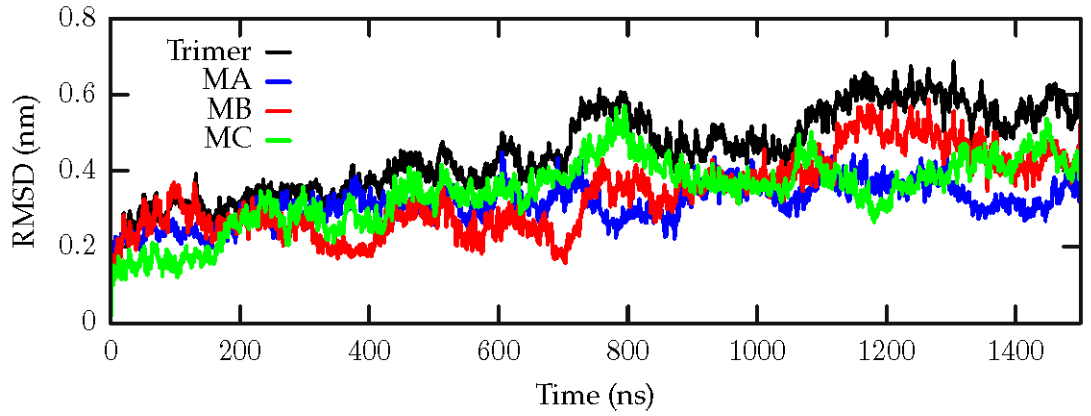

Supplementary Figure S2. Number of hydrogen bonds present in each monomer (MA, MB, and MC) as a function of the simulation time.

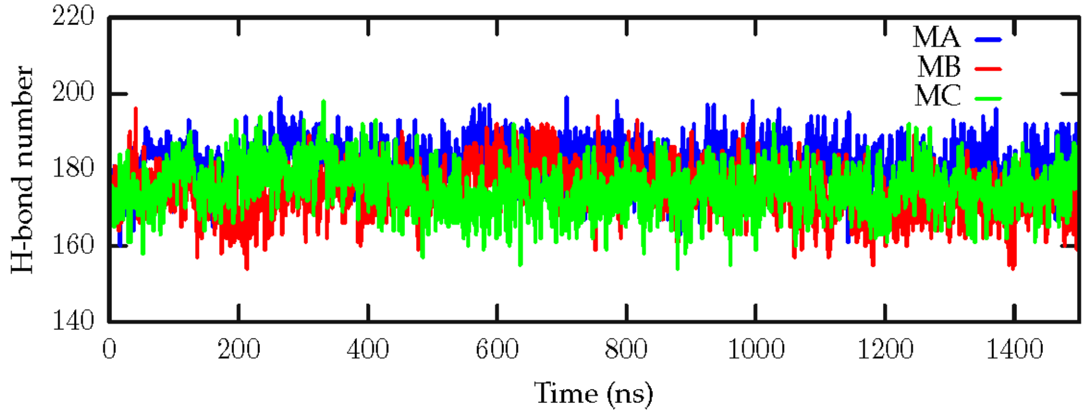

Supplementary Figure S3. Root-mean-square fluctuations calculated on the protein backbone of each monomer (MA, MB, and MC) averaged on the last 500 ns of MD simulation. The regions highlighted with orange boxes correspond to the portions of the two β-strands including residues E123 (β5) and R175 (β10).

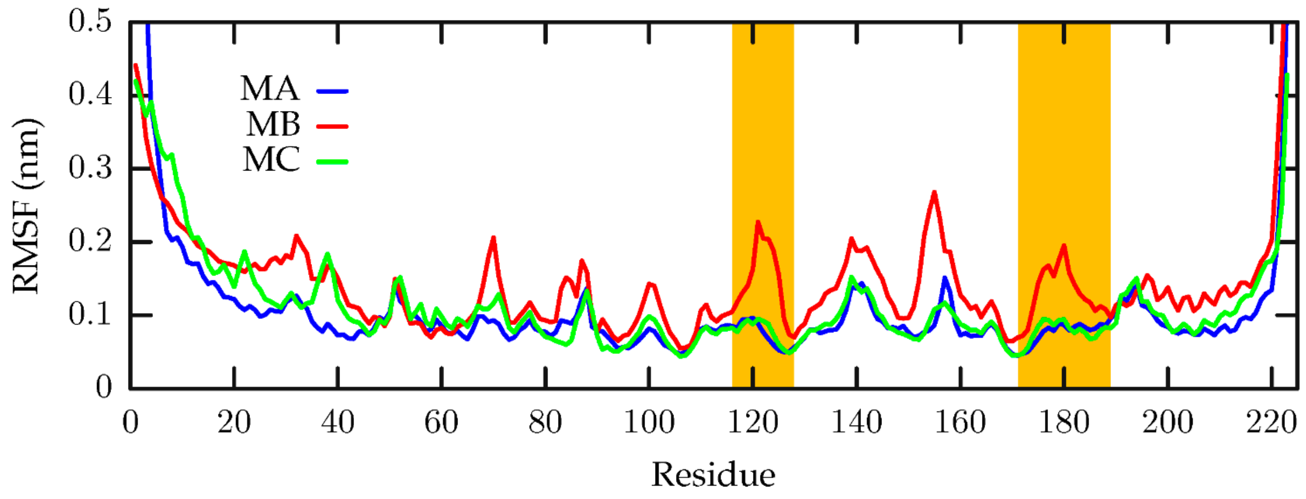

Supplementary Figure S4. Calculated energy of ligand binding to *Hs*σ1-R structure in coordinate file 5HK1.

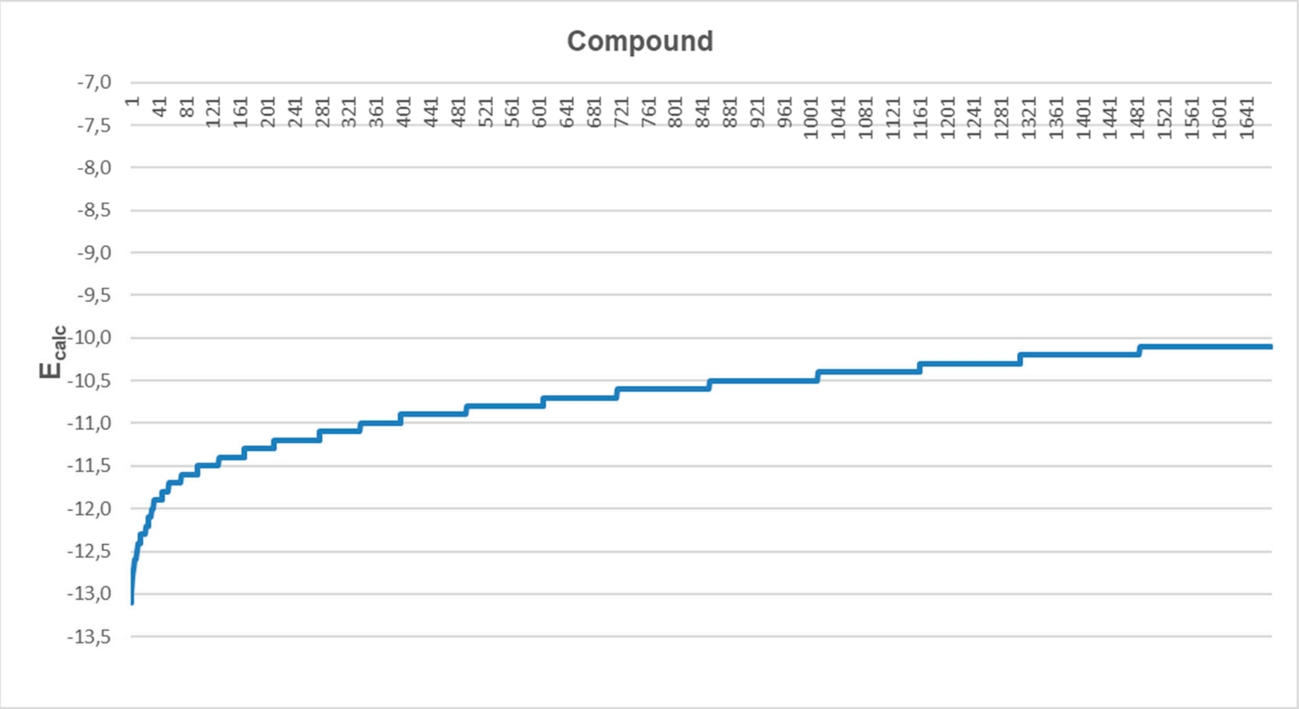

Supplementary Figure S5. Calculated energy of ligand binding to *Hs*σ1-R structure in coordinate file 6DK1.

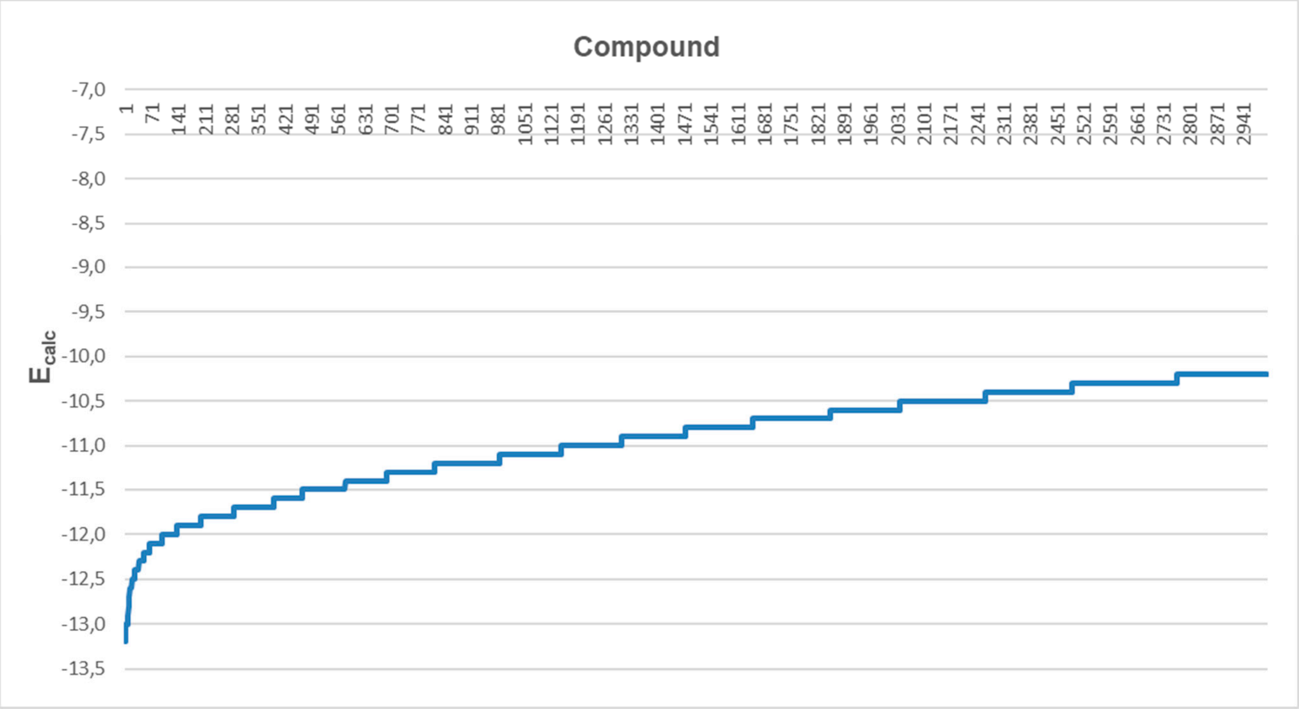

Supplementary Figure S6. Comparison between the calculated energy of ligand binding to *Hsσ*1-R structures in coordinate files 5HK1 and 6DK1.

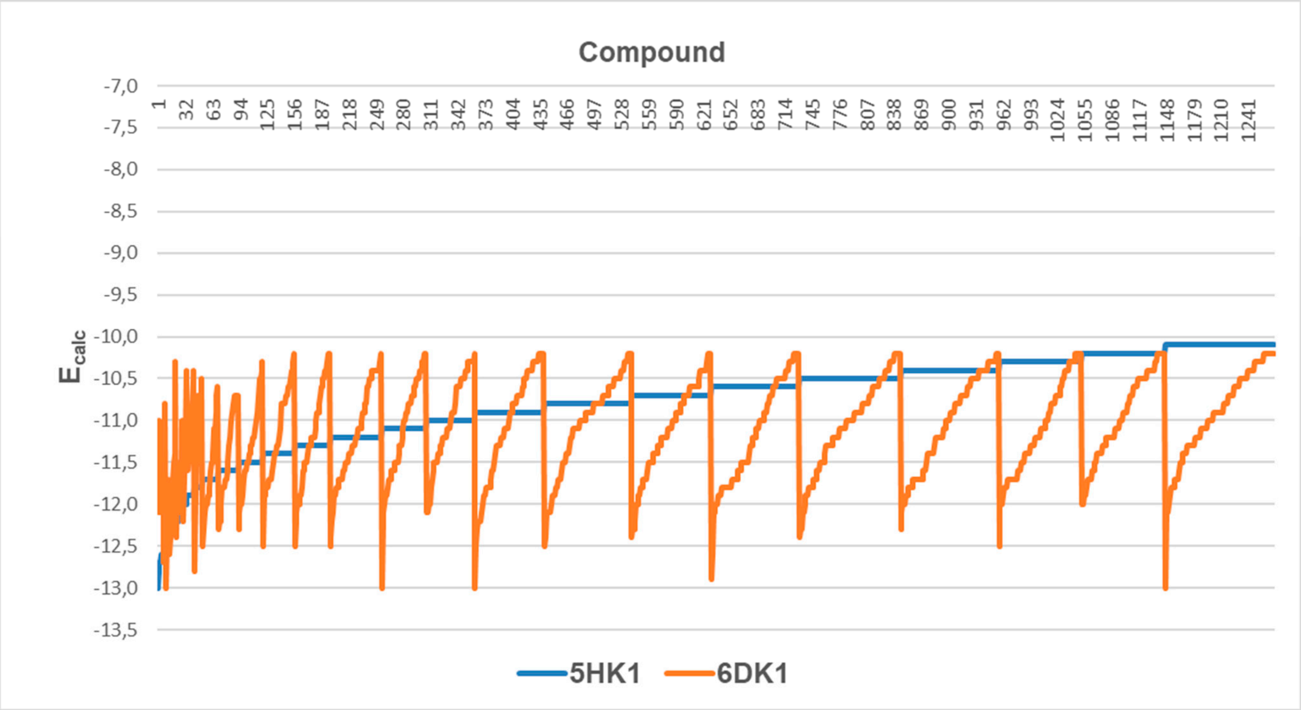

Supplementary Figure S7. Calculated energy of ligand binding to yeast ERG2 molecular model.

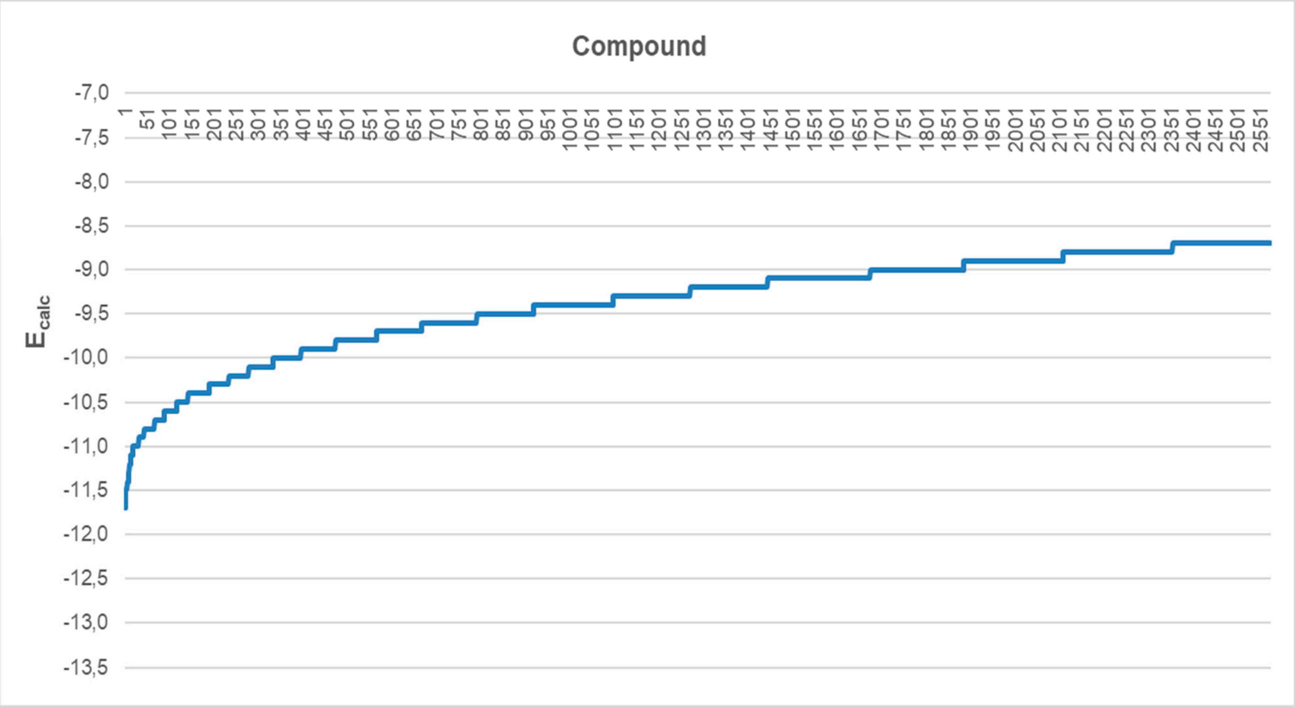

Supplementary Figure S8. Calculated energy of ligand binding to *Xl*σ1-R structure in coordinate file 7W2B.

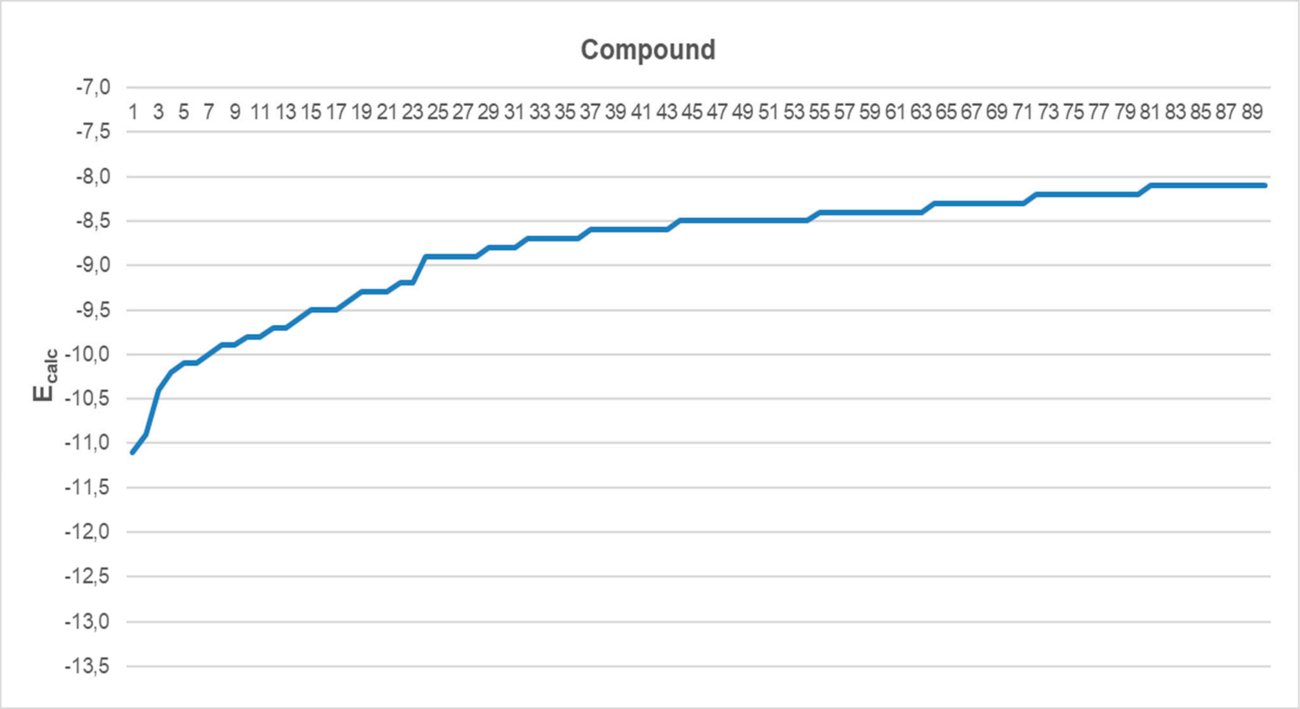

Supplementary Figure S9. Calculated energy of ligand binding to *Xl*σ1-R structure in coordinate file 7W2E.

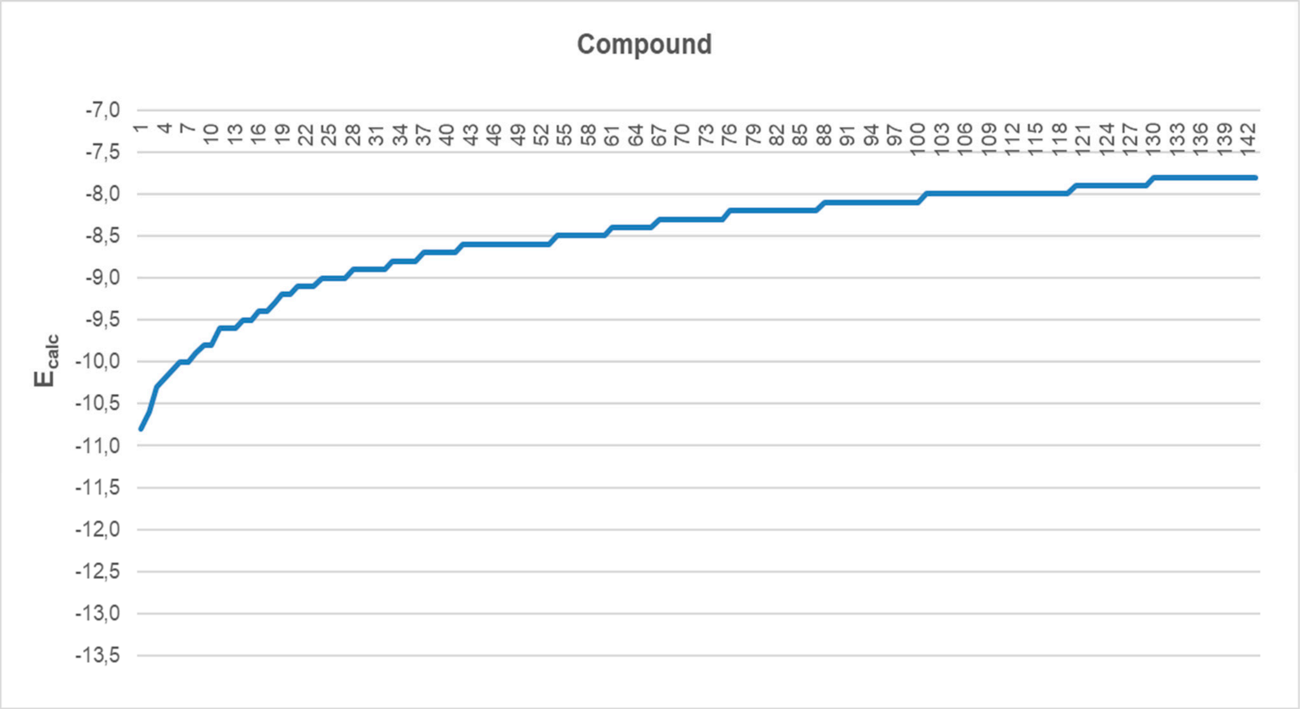

Supplementary Figure S10. Comparison between the calculated energy of ligand binding to *Xl*σ1-R structures in coordinate files 7W2B and 7W2E.

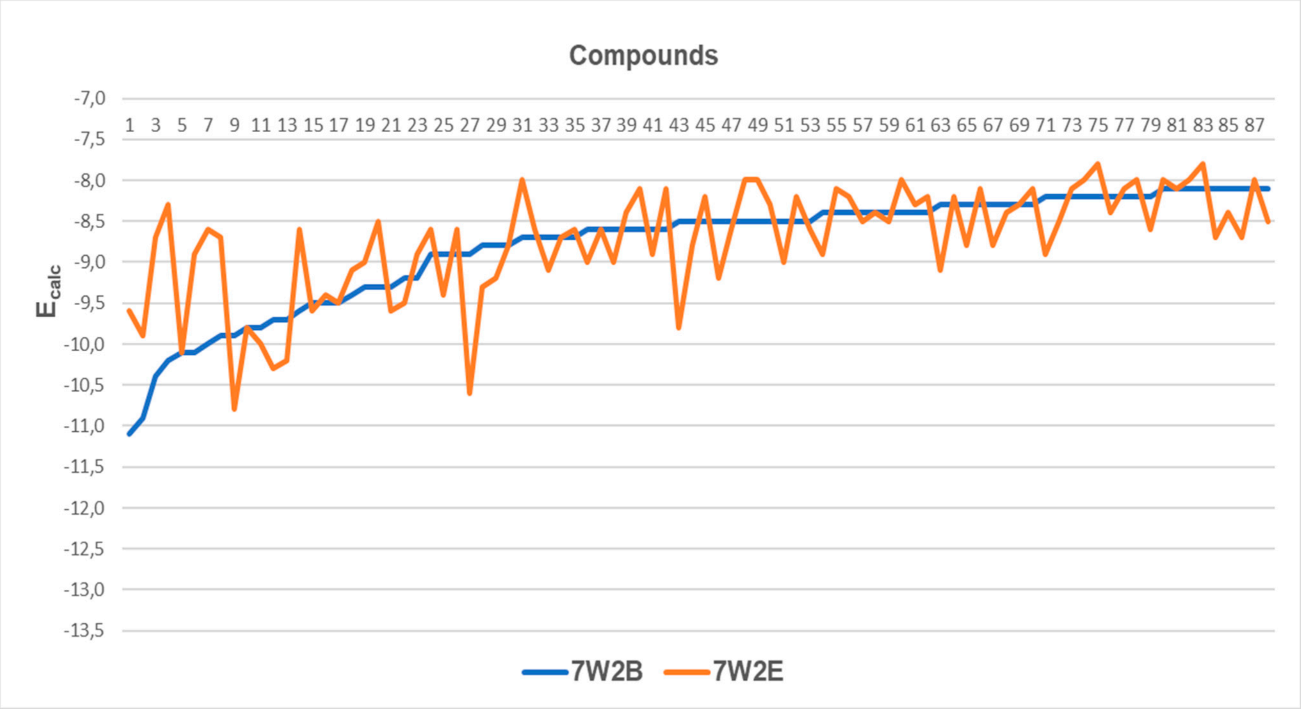

Supplementary Figure S11. Location of tryptophane residues in the 3D structure of *Hs*σ1-R in coordinate file 5HK1_C with respect to the ligand binding site. The protein main-chain is shown as ribbon and colored green. Side-chains of tryptophane residues are shown as sticks and colored by atom type: N, blue; C, white for the two residues in the proximity of the ligand binding site and orange for the others. The PD144418 ligand in complex with the protein is shown as sticks and colored by atom type: N, blue; O, red; C, yellow.

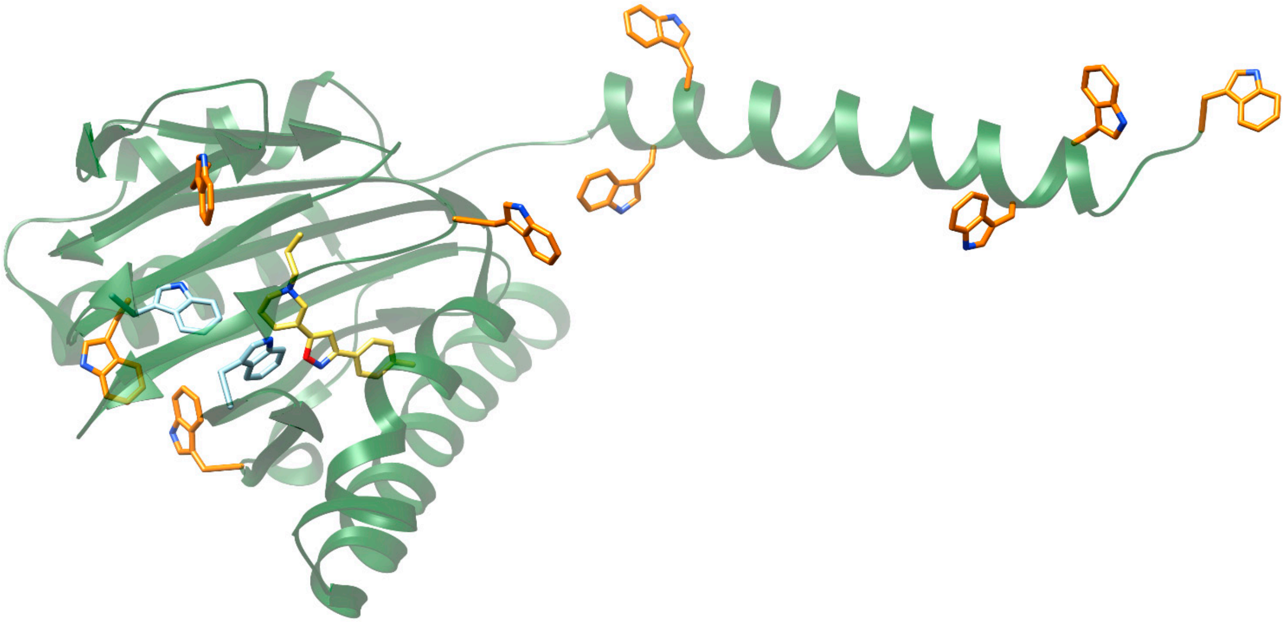

Supplementary Figure S12. (A) Calibration of the Superdex 200 size exclusion chromatography column. The x and y axes report the ratio between elution volume (V_e) and dead volume (V_0), and the logarithm of the molecular weight (log[MW]), respectively. The name and molecular weight of the proteins used to build the calibration curve are indicated. **(B)** Analytical size exclusion of purified *Hs*σ1R in LMNG/CHS detergent buffer on a Superdex 200 column. The x and y axes report the retention time and the absorbance at 280 nm, respectively. We obtained a peak at 13.9 min with a flow rate of 0.75 ml/min (V_0 = 8,07 ml).

A

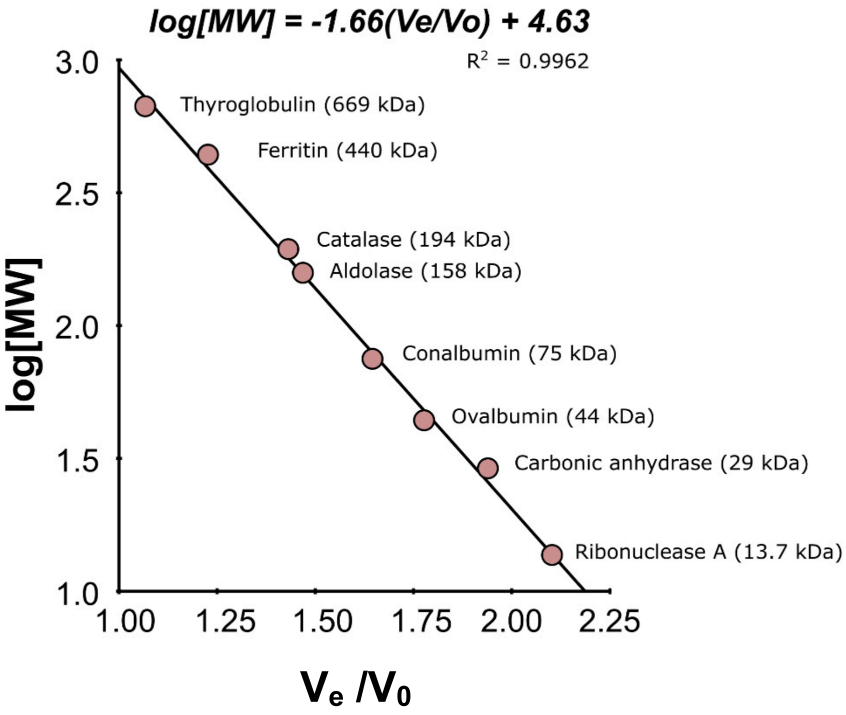

B

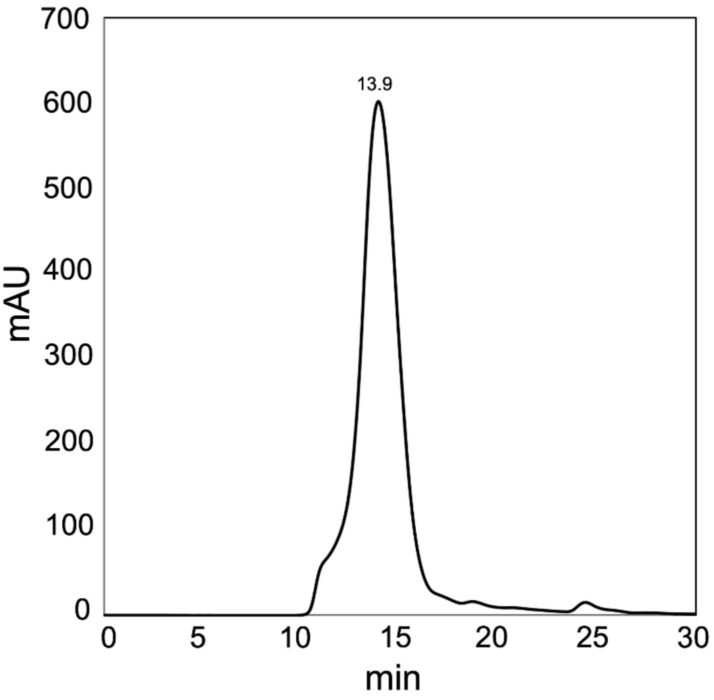

Supplement: Supplementary file 1 [file ijms-24-06367-s001.zip › Sigma1R_Supplementaries.pdf]
